# Application of a Low Transition Temperature Mixture for the Dispersive Liquid–Liquid Microextraction of Illicit Drugs from Urine Samples

**DOI:** 10.3390/molecules26175222

**Published:** 2021-08-28

**Authors:** Valeria Gallo, Pierpaolo Tomai, Valerio Di Lisio, Chiara Dal Bosco, Paola D’Angelo, Chiara Fanali, Giovanni D’Orazio, Ilaria Silvestro, Yolanda Picó, Alessandra Gentili

**Affiliations:** 1 Department of Chemistry, Sapienza University of Rome, 00185 Rome, Italy; gallo.1657510@studenti.uniroma1.it (V.G.); pierpaolo.tomai@uniroma1.it (P.T.); valerio.dilisio@uniroma1.it (V.D.L.); chiara.dalbosco@uniroma1.it (C.D.B.); p.dangelo@uniroma1.it (P.D.); ilaria.silvestro@uniroma1.it (I.S.); 2 Unit of Food Science and Nutrition, Department of Science and Technology for Humans and the Environment, Università Campus Bio-Medico di Roma, 00128 Rome, Italy; C.Fanali@unicampus.it; 3 Institute for the Biological Systems, National Research Council, 00015 Monterotondo, Italy; giovanni.dorazio@cnr.it; 4 Environmental and Food Safety Research Group, Desertification Research Centre (CIDE), CSIC-GV-UV, University of Valencia (SAMA-UV), 46113 Moncada, Spain; yolanda.pico@uv.es

**Keywords:** low transition temperature mixtures, deep eutectic solvents, dispersive liquid–liquid microextraction, drugs, high performance liquid chromatography, illicit drugs, urine, biological samples

## Abstract

The use of psychoactive substances is a serious problem in today’s society and reliable methods of analysis are necessary to confirm their occurrence in biological matrices. In this work, a green sample preparation technique prior to HPLC-MS analysis was successfully applied to the extraction of 14 illicit drugs from urine samples. The isolation procedure was a dispersive liquid–liquid microextraction based on the use of a low transition temperature mixture (LTTM), composed of choline chloride and sesamol in a molar ratio 1:3 as the extracting solvent. This mixture was classified as LTTM after a thorough investigation carried out by FTIR and DSC, which recorded a glass transition temperature at −71 °C. The extraction procedure was optimized and validated according to the main Food and Drug Administration (FDA) guidelines for bioanalytical methods, obtaining good figures of merit for all parameters: the estimated lower limit of quantitation (LLOQ) values were between 0.01 µg L^−1^ (bk-MMBDB) and 0.37 µg L^−1^ (PMA); recoveries, evaluated at very low spike levels (in the ng-µg L^−1^ range), spanned from 55% (MBDB) to 100% (bk-MMBDB and MDPV); finally, both within-run and between-run precisions were lower than 20% (LLOQ) and 15% (10xLLOQ).

## 1. Introduction

The fight against illicit drug use is still a current and central issue in all countries. As reported by the European Monitoring Center for Drugs and Drug Addiction, in the last year, it was estimated that more than 29% of European people have tried illicit drugs during their lifetime. Currently, drug consumption in Europe covers a wider range of substances than in the past, in line with poly-drug abuse, which is very common among both regular and occasional users. “New psychoactive substances” are analogues of well-known drugs such as cannabinoids, hallucinogens, and psychostimulants [1]. They include many synthetic cathinones (SCs), arylcyclohexylamines, phenethylamines, and tryptamines. Cathinone is a natural psychoactive compound occurring in the khat plant (*Catha edulis*), often referred to as a “natural amphetamine” due to their similar chemical structure (it is the β-ketone analogue of amphetamine) and behavioral effects. SCs were initially sold as “bath salts”, “plant food”, or “stain remover”, labeled “not for human consumption” or “for external use only” to hide their actual use and to circumvent statutory restrictions [2]; in this way, they could be easily purchased over the Internet or retail locations such as smart shops and smoke shops [1]. Initially, they were also known as “legal highs” due to their lawful status and were supposed to be innocuous alternatives to cocaine and amphetamine [3]. Because of this situation, in 2010, 3,4-methylenedioxypyrovalerone (MDPV) was one of the most widespread drugs in the United States [4]. These substances are now illegal, but new molecules with slight chemical modifications are continuously synthesized, with α-pyrrolidinoenanthophenone (*α*-PEP) as one of the last examples [4]. However, the problem is not limited to the SCs alone; for example, amphetamine derivatives and “ecstasy” (3,4-methylenedioxymethamphetamine, MDMA) are extremely abused drugs [5]. Many of these “new psychoactive substances” are especially consumed to enhance social and sexual experiences and because they are cheaper than cocaine; nevertheless, they can exert potentially toxic and lethal adverse effects, posing a grave threat to public health.

Currently, the main biological matrices used to verify the consumption of illicit substances are saliva, urine, blood, and hair [6], each of them with its own practical advantages and limitations. Among all, urine is convenient because large volumes can be sampled with a minimally invasive procedure; moreover, after their consumption, many drugs and metabolites are present in high concentrations for 2–3 days and remain stable in frozen urine [7]. Thus far, several analytical methodologies have been published for the determination of either conventional drugs of abuse or new psychoactive substances in urine [8,9,10]; conversely, their simultaneous analysis is still poorly explored. Most methods described for the extraction of illicit drugs from urine include dilution-and-shoot (DNS), solid phase extraction (SPE), liquid–liquid extraction (LLE), all of them applied directly or after a hydrolysis step with β-glucuronidase [8,9,10]. These sample pre-treatments are often followed by liquid chromatography-mass spectrometry (LC-MS) analysis, either when high sensitivity and selectivity are necessary because of a low (LLE) or no pre-concentration step (DNS) and/or when a confirmation analysis is required. Even if SPE-based procedures are the gold standard to treat urine, microextraction techniques are catching on due to their greater sustainability related to the minimal waste of organic solvents and many practical advantages. Among all the microextraction techniques, dispersive liquid–liquid microextraction (DLLME) stands out for its easiness, speediness, and high enrichment factor. This technique, based on a binary or ternary solvent system, involves the rapid injection of an extraction solvent and, if necessary, of a dispersing solvent in the aqueous sample; after mixing, which can be manual or supported by various methods (vortex, sonication, air bubbles) to favor the formation of a cloudy solution, the organic phase is taken with a micro-syringe, evaporated, reconstituted with a suitable solvent system, and finally analyzed chromatographically. Thus far, DLMME has widely been used for the analysis of environmental waters [11], while to a lesser extent to biological fluids such as saliva [12] and urine [13,14,15,16,17,18,19]. This pre-treatment technique is considered greener than LLE because it requires only a few hundred microliters of organic solvents. However, the most recent versions of DLLME see the replacement of organic solvents with last-generation “drinkable” solvents such as eutectic solvents (ESs) and low transition temperature mixtures (LTTMs). ESs, which include both deep (DES) and ideal (IES) eutectic solvents, are mixtures with a melting point lower than that of the individual solid components, while LTTM is a mixture exhibiting a glass transition [12,20,21,22]. IESs [23], DESs, and LTTMs are of special interest for analytical purposes, especially when liquid is at room temperature.

To the best of our knowledge, among the many variants in which this technique has been used, there are no applications of IES/DES/LTTM-based DLLME for the determination of illicit drugs from urine. The aim of this work was to apply a LTTM, composed of choline chloride:sesamol in a molar ratio 1:3 (ChCl:Ses, 1:3), as an extraction solvent during the DLLME of 14 drugs of abuse from urine samples. Mixtures of ChCl and Ses in different molar ratios (1:1, 1:2, 1:3, and 1:4) were prepared and characterized by differential scanning calorimetry (DSC) and infrared spectroscopy (IR), obtaining interesting results. Finally, the method was validated on the real matrix.

## 2. Results

### 2.1. Preparation and Characterization of Some ChCl:Ses Mixtures 

**Figure 1:**
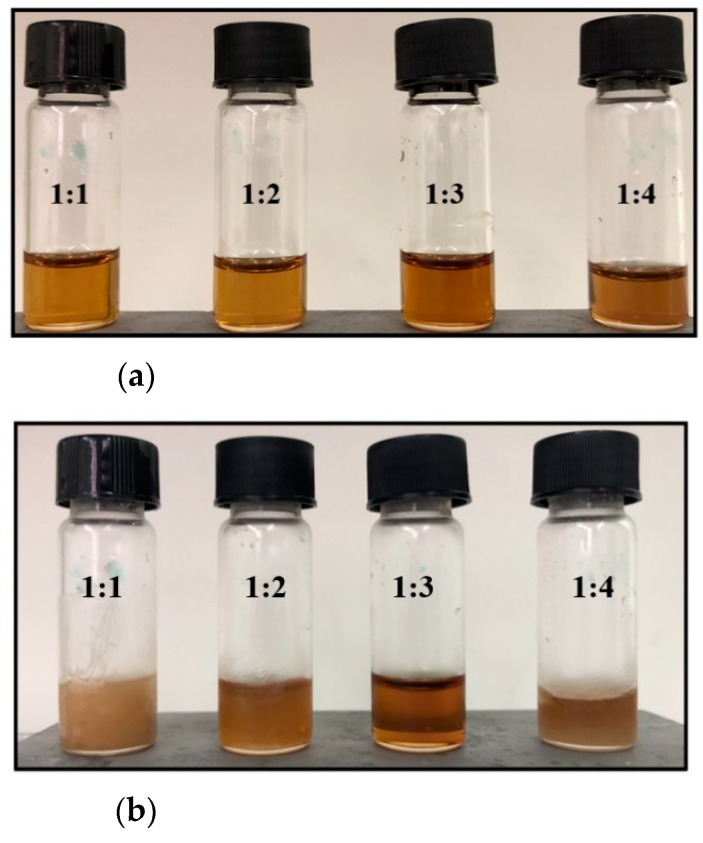
ChCl:Ses mixtures as prepared (**a**) and after storage at 4 °C for 24 h (**b**).

In order to prepare a green solvent that is liquid at room temperature, ChCl and Ses were selected as the hydrogen bond acceptor (HBA) and hydrogen bond donor (HBD), respectively, because of their physicochemical characteristics and for being natural, cheap, and low-toxicity compounds. After being mixed in different molar ratios (i.e. 1:1, 1:2, 1:3, 1:4) and heated at 50 °C for 10 min, the mixtures were cooled at room temperature (see Table S1 in the Supplementary Materials). At first glance, the freshly prepared eutectic mixtures (Figure 1a) appear stable in liquid form, although, after 24 h storage at 4 °C (Figure 1b), ChCl:Ses 1:3 is a unique composition that persists in the liquid state. The 1:1 mixture crystallizes completely, while the 1:2 and 1:4 mixtures appear as cloudy liquids, indicating the occurrence of partial crystallization. Indeed, ChCl:Ses with a molar ratio of 1:3 was selected as the extracting solvent in the following DLLME operations to have a stable liquid at sub-ambient temperature conditions (20 °C).

**Figure 2:**
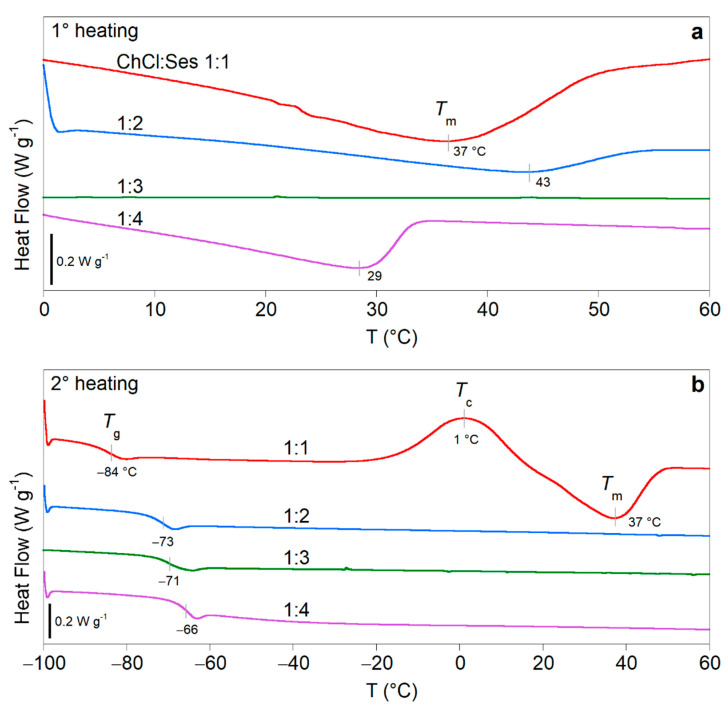
DSC heating traces of the ChCl:Ses 1:1, 1:2, 1:3, and 1:4 mixtures recorded at 10 °C/min, after storage at 4 °C for 24 h (**a**, first heating), and after in-situ cooling from 60 to −100 °C at −10 °C/min (**b**, second heating). The glass transition (Tg), recrystallization (Tc), and melting (Tm) temperatures are highlighted.

To obtain information about the thermal properties and molecular interactions of the four mixtures, they were characterized by DSC and IR spectroscopy. Figure 2 shows the DSC traces of the four mixtures acquired soon after thermal treatment at 4 °C for 24 h (first heating, Figure 2a) and after in-situ cooling at 10 °C/min (second heating, Figure 2b). During the first heating, the 1:1, 1:2, and 1:4 ChCl:Ses mixtures showed endothermic peaks at Tm = 37, 43, and 29 °C, indicating the melting temperatures of the crystals formed during storage at 4 °C. As expected, the 1:3 trace did not show any thermal transition. During the cooling, all mixtures remained in the amorphous state by undergoing a liquid–glass transition (data not shown). In the second heating, glassy mixtures evolved toward the liquid state at the glass transition temperature, which was observed as a step in the heat flow at Tg = −84, −73, −71, and −66 °C for the 1:1, 1:2, 1:3, and 1:4 ChCl:Ses mixtures, respectively. Moreover, the 1:1 composition also exhibited a recrystallization at Tc = 1 °C, followed by the melting at the same temperature of the previous heating step (Tm = 37 °C).

The calorimetric characterization of the ChCl:Ses systems highlights that the 1:1, 1:2, and 1:4 ChCl:Ses mixtures, if treated at sub-ambient conditions, were able to crystallize. The melting temperatures of such crystal phases were between 29 and 43 °C, far below the melting transition of their single components (Tm (ChCl) estimated 324 °C [24] and Tm(Ses) = 62–65 °C). On the other hand, at least in the current experimental conditions, the 1:3 ChCl:Ses did not exhibit crystallization/melting phenomena, therefore, it can be classified as an LTTM.

**Figure 3:**
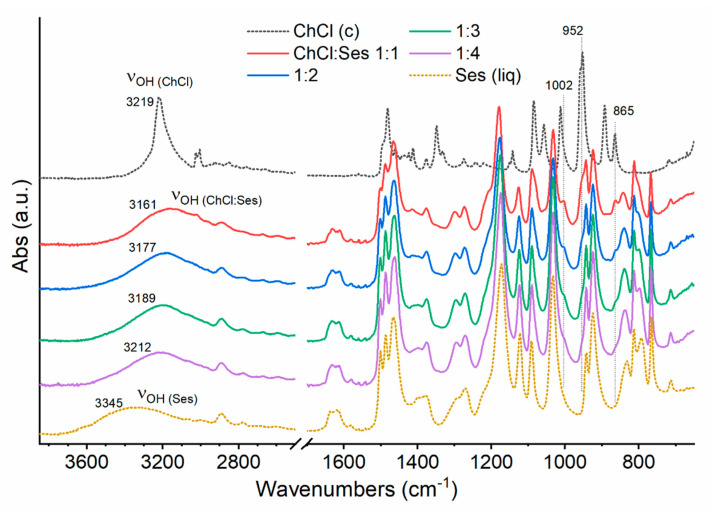
ATR-FTIR spectra of crystalline ChCl (black dotted line), liquid Ses (yellow dotted line), and ChCl:Ses eutectic mixtures (solid lines) with ChCl:Ses 1:1, 1:2, 1:3, and 1:4, acquired at room temperature.

Figure 3 shows the ATR-FTIR spectra of ChCl:Ses mixtures 1:1, 1:2, 1:3, and 1:4, compared with those of crystalline ChCl (c) and liquid Ses (liq). In general, the spectral features of the ChCl:Ses mixtures mostly resemble those of liquid Ses in the 1700–650 cm^−1^ region, comprising the C=C stretching at 1620 cm^−1^, the C–H bending region between 1500 and 1400 cm^−1^, the C-O stretching bands between 1300 and 1000 cm^−1^, and the ring deformation band at 765 cm^−1^. Moreover, weak bands associated with liquid ChCl arose in the ChCl:Ses spectra at 1002, 952, and 865 cm^−1^, which increased with increasing ChCl content. Major spectral differences were observed in the OH stretching region (ν_OH) between 3600 and 3000 cm^−1^. In fact, the shape and position of the OH band was greatly affected by intra- and inter-molecular interactions existing in the system. In particular, the absorption band was red-shifted and sharp if strong and spatially ordered hydrogen bonds were present between the hydroxylic groups, as in the case of crystalline ChCl (ν_(OH(ChCl)) = 3219 cm^−1^). On the other hand, broad and blue-shifted bands arose from disordered and weakly hydrogen bonded systems, as occurred in liquid Ses (ν_(OH(Ses)) = 3345 cm^−1^). The OH stretching absorption of liquid ChCl:Ses mixtures can be considered as the sum of the stretching bands of both the hydroxylic groups of ChCl and Ses. However, a broad unresolved band was observed for the liquid mixtures, inferring that the OH groups of the two compounds absorbed at approximately the same wavenumber. Moreover, the broad absorption of the eutectic mixtures highlights the absence of any long-range order in the hydrogen bond network. Noteworthy, the ν_(OH(ChCl:Ses)) stretching bands for the 1:1, 1:2, 1:3, and 1:4 ChCl:Ses mixtures were located at 3161, 3177, 3189, and 3212 cm^−1^, respectively. In all cases, the OH absorption was red-shifted with respect to their single components. Indeed, the average strength of intermolecular interactions, that is inversely proportional to the band wavenumber location, was the maximum for the 1:1 ChCl:Ses composition, and slightly decreased with the increase in Ses content. As shown by the red-shifting of the OH stretching bands, the average intermolecular interactions between ChCl and Ses molecules were stronger in the whole explored composition range compared to those existing both in the crystal structure of ChCl and in liquid Ses.

### 2.2. Optimization of the DLLME Procedure

In this work, the procedure by Gallo et al. [22], developed to isolate pesticides from urine samples, was conveniently modified to maximize the recovery of the selected illicit drugs. All optimization experiments were performed in triplicate using 5 mL of diluted urine per test (3 mL of human urine + 2 mL of Milli-Q water), spiked with the target analytes at 1 µg L^−1^. As previously verified [22], the dilution of a urine sample is a simple strategy to reduce the matrix effect and to improve the phase separation during the DLLME procedure. The initial protocol was as follows: the dilute urine samples were treated using 100 µL of LTTM as the extracting solvent and 400 µL of ethyl acetate as the dispersing solvent; the cloudy solution was vortex-mixed for 2 min and, after centrifugation, the settled phase was taken and 2 µL was injected into the LC-MS system. Under these conditions, recoveries were unsatisfactory because they were below 40%. 

**Figure 4:**
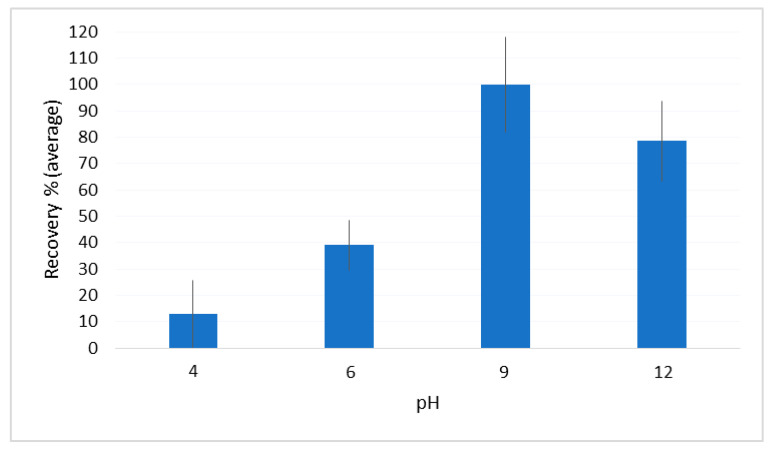
The pH effect on the analyte recovery.

Given the neutral-basic nature of the target compounds (pKa 10–13), the effect of the sample pH was investigated by conducting another three series of experiments by adjusting the pH of diluted urine samples at 4 with HCl, and at 9 and 12 with NaOH. Figure 4 resumes the recoveries, averaged on all the analytes, obtained for the different pH values.

Compared with the result obtained for the diluted urine aliquots whose pH was not corrected (it was around 6), the extraction carried out at acidic pH caused a sharp decrease in the yield, which did not exceed 10%. On the other hand, quantitative recoveries were obtained at pH 9, while more alkaline conditions caused a slight drop (~78%).

To evaluate the effect of the ionic strength on recoveries, urine samples containing different concentrations of NaCl (5, 25, 50, and 100 mg mL^−1^) were prepared and extracted (also in this case, three replicates per condition). Salt addition did not improve the recoveries, probably because the addition of NaOH, made to adjust the sample pH, also has the further advantage of adjusting the ionic strength favoring the separation phase and the analyte transfer toward the microdroplets of the extracting solvent in the cloudy solution.

### 2.3. Method Validation

Quantitative analysis was performed building matrix-matched calibration curves. To this end, nine 5-mL aliquots of diluted blank urine were spiked with increasing concentrations of the target analytes (LLOQ level and 1, 15, 30, 45, 60, 75, 100 µg L^−1^) and extracted according to the protocol described in Section 3.6. For each analyte, the peak area was plotted versus the spike level (μg L^−1^) by applying the least-square method to find the best fit for each dataset (y = a + bx as regression model). Table 1 lists the linear regression parameters. As can be seen, the determination coefficients (R^2^) were greater than 0.92 for all the analytes, which is a good result considering that the curves were built in the matrix, spiking the calibrators’ pre-extraction. Usually, R^2^ greater than 0.90 guarantees the adequacy of the fitted model [25].

Urinary creatinine is a by-product of muscle metabolism, whose excretion is independent of urine flow. Due to this peculiarity, it is used to correct for analyte concentration in urine [26]. Since creatinine concentration in urine is very high (up to 0.4–3.0 g L^−1^ [27]), its determination in real samples was estimated by diluting 50 µL of urine with Milli-Q water in a 50-mL volumetric flask; then, a 2-μL volume was directly injected for the HPLC-MS analysis. Considering the high dilution ratio applied (1:1000), the matrix effect was negligible [22] and the concentration of creatinine in real samples was calculated by means of external calibration. To this end, five 50-µL aliquots of Milli-Q water (instead of 50-µL aliquots of urine) were diluted in 50-mL volumetric flasks and spiked with the creatinine standard solution to obtain the following concentrations: 0.0625 mg L^−1^, 0.125 mg L^−1^, 0.625 mg L^−1^, 1.25 mg L^−1^, and 2.5 mg L^−1^. Such concentrations corresponded to g L^−1^ spike levels in the pure urine (in fact the dilution ratio was 1:1000). Table 1 shows the calibration curve and *R*^2^ for creatinine.

For each analyte, LLOQ was calculated as the spike level able to provide a signal-to-noise ratio of 5 (five replicates). To this end, 5- mL aliquots of dilute urine (see Section 3.6) were spiked pre-extraction with the analytes at decreasing concentrations until meeting the described requirements. Once the LLOQ was experimentally verified, its average value was calculated preparing five replicates. As can be seen in Table 2, the LLOQ values ranged between 0.01 µg L^−1^ (bk-MMBDB) and 0.37 µg L^−1^ (PMA).

To calculate recovery and within-run precision, five 5-mL aliquots were spiked with the analytes’ pre-extraction at two concentration levels corresponding to LLOQ and 10xLLOQ; another aliquot was spiked post-extraction with the same nominal concentrations. As shown in Table 2, the recoveries spanned between 55% (MBDB) and 100% (bk-MMBDB and MDPV), depending on the spike level. 

The enrichment factor (EF) was calculated according to the following equation:

(1) $$EF=\frac{C_{analyte\;in\;the\;final\;extract}}{C_{analyte\;in\;the\;urine\;sample}}$$

The procedure was able to reach EFs varying from 17.7 to 28.4 (under the established extraction conditions, the maximum achievable is 30) (see Table 2).

The within-run precision, defined as the relative standard deviation (RSD), was in the range of 8–18% (LLOQ) and 4–10% (10xLLOQ) (Table 2). The between-run precision was evaluated as the RSD of three different analytical sessions, calculated at the same spike levels (5-replicates per analytical sessions); its values were equal to or less than 15% (10LLOQ) and 20% (LLOQ). 

### 2.4. Comparison with Previous DLLME-Based Methods 

Table 3 resumes the main figure of merits of some recent DLLME-based methods with some analytes in common with this work. As far as LOD is concerned, our method provides much lower values (ng L^−1^ vs. µg L^−1^) imputable to the higher sensitivity of the triple quadrupole detection system. Regarding recovery and precision, our method showed an analogous performance to the others [13,14,15,16,17], with the difference that we evaluated these parameters by applying significantly lower spike levels, as can be seen in Table 2; this could also explain the lower yield obtained for MDBD. 

Concerning extraction time, our procedure is as rapid as the others, but it is safer for the operator and has a minimal environmental impact due to the use of this specific neoteric solvent. Finally, using the ChCl:Ses 1:3 mixture, the evaporation step can be skipped, and the direct injection in the chromatographic system makes the procedure leaner.

Sensitivity, good recoveries, and the other advantages make our procedure suitable for routine monitoring for which, besides saving time and money, also greenness has considerable importance.

## 3. Experimental

### 3.1. Chemicals, Materials, and Solutions

α-pyrrolidinopentiophenone (α-PVP), dibutylone (*bk*-MMBDB), bufotenine (BUF), codeine (COD), 3,4-methylenedioxymethamphetamine (MDMA), ephedrine (EPH), heroin (HER), ketamine (KET), *N*-methyl-1-(3,4-methylenedioxyphenyl)-2-butanamine (MBDB), 3,4-methylenedioxypyrovalerone (MDPV), 3′,4′-methylendioxy-α-pyrrolidinopropiophenone (MDPPP), 4′-methyl-α-pyrrolidinobitiophenone (MPBP), naphyrone (NAPH), para-methoxyamphetamine (PMA), and creatinine were bought from Cerillant (Austin, TX, USA). All chemicals had a purity greater than 99%. Table S2 in the Supplementary Materials summarizes the physicochemical characteristics of the selected compounds.

Acetonitrile, methanol, toluene, ethyl acetate, choline chloride (ChCl), sesamol (Ses), NaCl, and NaOH were purchased from Merck Life Science S.r.l. (Milan, Italy). Each stock solution was prepared by dissolving a weighed standard amount of the compound in methanol to obtain a concentration of 1 mg mL^−1^. Working standard solutions were obtained by diluting the individual stock solutions at the suitable concentrations for the optimization and validation experiments. When unused, all solutions were stored in the darkness at −18 °C. 

### 3.2. Urine Samples

Urine samples were taken daily from healthy voluntary donors from our research group (both sexes, aged between 20 and 60 years). A pool of urine from the different donors (~50 mL) was also collected and then subsampled for use in method optimization and validation. All urine samples were iced and stored at −18 °C until analysis.

### 3.3. Preparation of ChCl:Ses 1:3 Mixture

The ChCl:Ses 1:3 mixture was prepared according to the procedure developed in a previous work [22]. Briefly, in the protocol that was applied, ChCl was dried in a muffle oven at 80 °C for 24 h. Afterward, 1.000 g of ChCl and 2.983 g of Ses were quickly weighed in a 25-mL weighing bottle and blended with a spatula. After closing the weighing bottle, the solid mixture was heated at 50 °C under magnetic stirring until the complete formation of a viscous amber liquid (~5 min). Then, the liquid mixture (~3 mL) was allowed to cool at room temperature and could be utilized to perform about 30 extractions. When unused, the LTTM was kept at room temperature.

### 3.4. Differential Scanning Calorimetry

Thermograms were acquired by using a Mettler Toledo 822e heat flux calorimeter equipped with an FRS-5 sensor and liquid nitrogen cooling. The oven was purged with dry nitrogen with a flow rate of 30 mL/min. Samples were prepared by weighting about 5–8 mg of liquid ChCl:Ses mixture in a 40 µL aluminum crucible and quickly sealed. Samples were kept at 4 °C for 24 h before the scan to induce crystallization. Calorimetric traces were performed at a heating/cooling rate at 10 °C min^−1^, with a temperature program comprising of a first heating from 0 to 60 °C, a cooling up to −100 °C, and a second heating between −100 °C to 60 °C.

### 3.5. Infrared Spectroscopy

FTIR spectra of the Ses (liq), ChCl (c), and ChCl:Ses mixtures were acquired in attenuated total reflectance mode (ATR-FTIR) by means of a Nicolet 6700 FTIR by Thermo Fisher Scientific, equipped with a Specac Golden Gate ATR accessory. Spectra were collected in the 4000–650 cm^−1^ spectral range by co-adding 100 scans at a resolution of 4 cm^−1^. The spectrum of Ses (liq) was obtained by melting the crystalline compound at 70 °C directly on the ATR crystal, and by acquiring the spectrum at room temperature before the crystallization occurred.

### 3.6. Extraction Procedure

In this paper, the procedure from a previous work [22] was suitably modified according to the nature of the target analytes to both maximize the extraction yield and improve the phase separation. A 3-mL urine sample was spiked with 200 µL of 0.2 mM NaOH to adjust its pH to 10, and then centrifuged at 10,000 rpm for 5 min. The supernatant was taken, placed in a 15-mL centrifuge tube, and diluted with 2 mL of Milli-Q water to obtain a total volume of 5 mL. The rapid sequential injection of ChCl:Ses 1:3 (100 μL) and ethyl acetate (400 μL), followed by vortexing for 1 min, generated a fine cloudy solution. Upon centrifuging at 10,000 rpm for 10 min, ChCl:Ses 1:3 settled at the bottom of the tube and could be withdrawn with a micro-syringe (100 μL). Finally, the extract was transferred to a vial and a 2-μL volume was injected for the HPLC-MS analysis.

Analyte concentrations were normalized toward the creatinine concentration as follows (Equation (1)):

(2) $$C_{normalized}=\frac{C_{analyte}\left(\mu g\;L^{-1}\right)}{C_{creatinine\;}\left(\mu g\;L^{-1}\right)}$$

### 3.7. HPLC-MS Analysis

Liquid chromatography was performed with an Agilent 6410 UHPLC system (Agilent, Waldbronn, Germany). The analyte separation was obtained on a Phenomenex Kinetex C18 column (1.7 μm, 50 2.1 mm) kept at room temperature. The mobile phase was water (**A**) and methanol (**B**), both containing ammonium formate (10 mM). The elution was performed as follows with a flow rate of 0.300 mL min^−1^: 0–7 min, 10–40% B; 7–13 min, 40–60% B; 13–20 min, 60–95% B.

A triple quadrupole mass spectrometer Agilent 1260 UHPLC (Agilent, Waldbronn, Germany), equipped with an electrospray source operating in positive ionization (capillary voltage at 4000 V), was used as a detector for the identification and quantification of the analytes. Nitrogen was used as a curtain gas, fragmentation gas, and gas drying (flow = 11 L min^−1^). The heater of the drying gas was set at 300 °C.

**Figure 5:**
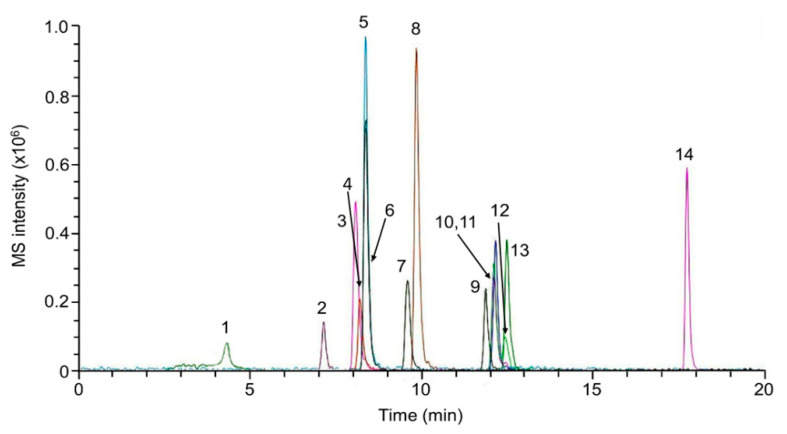
HPLC-MRM of a working standard solution (5 ng injected). Number of peak identification: 1. BUF, 2. COD, 3. MDMA, 4. MDPPP, 5. PMA, 6. EPH, 7. bk-MMBDB, 8. MBDB, 9. HER, 10. MPBP, 11. *α*-PVP, 12. KET, 13. MDPV, 14. NAPH.

Table 1 lists the LC-MS parameters useful for the target analytes identification. Figure 5 shows a LC-MRM chromatogram of a working solution in methanol (1 ng μL^−1^; 5 μL injected).

### 3.8. Method Validation

The method validation was carried out following the main FDA guidelines for the bioanalytical method validation [28]. Both quantitative and qualitative parameters were evaluated including recovery, enrichment factor, within-run and between-run precision, LLOQ, sensitivity, linearity, and selectivity. The method validation was performed in matrix using a pool of urine from the different donors (see Section 3.2).

## 4. Conclusions

ESs are considered green alternatives to classical molecular solvents for their negligible toxicity, low environmental impact, and low vapor tension. Their use continues to increase due to the flexibility of their formulation and the consequent possibility of obtaining a solvent system with the desired properties. In this paper, ChCl:Ses 1:3 was identified as a LTTM and used for the DLLME extraction of 14 illicit drugs from urine. The developed procedure displays all the advantages of a microextraction technique merged with those arising from the use of an ES. Due to the basic nature of the analytes, the adjustment of the pH sample at alkaline values was a key factor to increase recovery yields substantially. Although DLLME is typically applied to treat water samples, it has also been proven to be suitable for urine pre-treatment. Compared with other methods from the literature, our procedure showed similar extractive yields but at much lower spike levels, a minimal cost for single extraction, and a marginal impact on the environment and operator health. These are all strong points that make it very appealing for biomonitoring. Moreover, the ChCl:Ses 1:3 mixture might also exhibit antioxidant properties for the presence of Ses and, in such an eventuality, it could be useful for the extraction and preservation of analytes that are sensitive to photo-oxidation. 

## Figures and Tables

**Table 1:** Linear regression parameters.

Analyte	Regression Equation ^a^		R^2^
	b ± s_b_t_(0.05;6)_	a ± s_a_t_(0.05;6)_	
BUF	93.31 ± 0.29	118.81 ± 0.18	0.9931
COD	87.19 ± 0.72	273.95 ± 0.82	0.9624
MDMA	3241.30 ± 0.91	−7218.30 ± 0.55	0.9269
MDPPP	10.91 ± 0.40	50.29 ± 0.36	0.9572
PMA	74.02 ± 0.99	390.06 ± 0.97	0.9601
EPH	2731.70 ± 0.51	−5620.20 ± 0.63	0.9566
bk-MMBDB	305.42 ± 0.46	1237.20 ± 0.25	0.9802
MBDB	601.06 ± 0.98	1396.10 ± 3.04	0.9680
HER	205.63 ± 0.3	1046.8 ± 0.95	0.9611
MPBP	102.01 ± 0.6	816.04 ± 0.97	0.9597
α-PVP	38.31± 0.44	357.71 ± 0.20	0.9590
KET	465.22 ± 0.97	1346.40 ± 0.48	0.9539
MDPV	217.88 ± 0.76	679.06 ± 0.36	0.9869
NAPH	42.75 ± 0.48	349.17 ± 0.88	0.9783
Creatinine	2236.02 ± 5.03	77.90 ± 0.81	0.9988

^a^ Mean of six independent analyses.

**Table 2:** LLOQ, enrichment factor, recovery, precision, and accuracy.

Analyte	LLOQ (µg L^−1^)	Enrichment Factor ^a^	Recovery ^b^ %		Within-Run Precision ^b^ (RSD, %)	
			Spike Levels		Spike Levels	
			LLOQ	10xLLOQ	LLOQ	10xLLOQ
BUF	0.03	29.1	97	97	15	6
COD	0.02	22.5	71	79	14	11
MDMA	0.12	23.1	76	78	12	5
MDPPP	0.25	27.0	88	92	13	6
PMA	0.37	28.2	92	96	18	10
EPH	0.33	24.0	68	92	8	4
bk-MMBDB	0.01	28.4	89	100	18	10
MBDB	0.07	17.7	55	63	13	8
HER	0.07	21.3	63	79	11	7
MPBP	0.14	25.8	78	94	15	6
α-PVP	0.11	23.9	67	92	8	8
KET	0.09	28.1	89	98	9	6
MDPV	0.01	26.7	80	100	12	10
NAPH	0.02	26.6	80	97	13	6

^a^ The enrichment factor has been reported as mean values of data obtained for spiking levels at LLOQ and 10xLLOQ; ^b^ Recovery, precision, and accuracy were calculated preparing 5 replicates at each spike level.

**Table 3:** Comparison of the main figures of merit of some recent DLLME-based methods aimed at the extraction of illicit drugs from urine.

Method (Common Analytes)	Common Analytes	Enrichment Factor	Recovery %	Precision %	LOD (µg L^−1^)	Type and Volume of Solvents	Extraction Time (min)	Reference
DLLME-GC-MS	KET	Not provided	86–93 (20, 200 µg L^−1^ spike level)	5–11.7	1.25	Extr ^a^: protonated *N*,*N*-dimethylcyclohexylamine (400 µL) + NaOH 6M (400 µL)	~15	[13]
DLLME-GC/MS	KET	204	91	3.4	0.91	Pretreatment to adjust pH Extr ^a^: carbon tetrachloride; 30 µL Disp ^b^: EtOH; 500 µL	~15	[14]
Ionic liquid-based DLLME-capillary electrophoresis	EPH KET	28.8 16.9	86–90 79–82 (1–2 mg L^−1^ spike level)	6.8 6.8	15 30	Pretreatment with methanol and NaOH; Extr ^a^: [BMIM]PF6; 40 µL Disp ^b^: acetonitrile; 350 µL	~20	[16]
DLLME-GC/MS	KET MDMA MDBD *α*-PVP MDPV	Not provided	112 102 92 116 111 (50 µg L^−1^ spike level)	1.6 1.0 6.7 9.7 13.5	5 2 5 5 10	Pretreatment with methanol, NaOH, Na Cl; derivatization with hexyl chloroformate; Extr ^a^: Chloroform; 100 µL Disp ^b^: methanol; 250 µL	~20	[17]
DLLME-HPLC/MS	KET MDMA MDBD *α*-PVP MDPV	28.1 23.1 17.7 23.9 26.7	89–98 76–78 55–63 67–92 80–100 (LLOQ spike level, see Table 2)	6–9 5–12 8–13 8 10–12	0.054 0.072 0.042 0.066 0.006	Pretreatment with NaOH to adjust pH Extr ^a^: ChCl:Ses 1:3; 100 µL Disp ^b^: ethyl acetate; 400 µL	~15	This work

^a^ Extr: extraction solvent; ^b^ Disp: dispersing solvent.

## Data Availability

The data is not available.

## Supplementary Materials

The following are available online, Table S1: Structures, exact masses, and main physicochemical characteristics of the selected illicit drugs, Table S2: Conditions for the preparation of some mixtures based on choline chloride and sesamol.

## Sample Availability

The analyzed urine samples are not available from the authors.

## References

[1] Riley A.L., Nelson K.H., To P., López-Arnau R., Xu P., Wang D., Wangc Y., Shen H.-W., Kuhn D.M., Angoa-Perez M. (2020). Abuse potential and toxicity of the synthetic cathinones (i.e.,“Bath salts”). Neurosci. Biobehav. Rev..

[2] Schneir A., Ly B.T., Casagrande K., Darracq M., Offerman S.R., Thornton S., Smollin C., Vohra R., Rangun C., Tomaszewski C. (2014). Comprehensive analysis of “bath salts” purchased from California stores and the internet. Clin. Toxicol..

[3] Spiller H.A., Ryan M.L., Weston R.G., Jansen J. (2011). Clinical experience with and analytical confirmation of “bath salts” and “legal highs” (synthetic cathinones) in the United States. Clin. Toxicol..

[4] German C.L., Fleckenstein A.E., Hanson G.R. (2014). Bath salts and synthetic cathinones: An emerging designer drug phenomenon. Life Sci..

[5] Palamar J.J., Salomone A., Gerace E., Di Corcia D., Vincenti M., Cleland C.M. (2017). Hair testing to assess both known and unknown use of drugs amongst ecstasy users in the electronic dance music scene. Int. J. Drug Policy.

[6] Allen K.R. (2011). Screening for drugs of abuse: Which matrix, oral fluid or urine?. Ann. Clin. Biochem..

[7] Rivier L. (2000). Techniques for analytical testing of unconventional samples. Best Pract. Res. Clin. Endocrinol..

[8] Hegstad S., Hermansson S., Betnér I., Spigset O., Falch B.M.H. (2014). Screening and quantitative determination of drugs of abuse in diluted urine by UPLC–MS/MS. J. Chromatogr. B.

[9] Grueninger D., Englert R. (2011). Determination of the amphetamine-like designer drugs methcathinone and 4-methylmethcathinone in urine by LC-MS/MS. Annal. Toxicol. Anal..

[10] Concheiro M., Anizan S., Ellefsen K., Huestis M.A. (2013). Simultaneous quantification of 28 synthetic cathinones and metabolites in urine by liquid chromatography-high resolution mass spectrometry. Anal. Bioanal. Chem..

[11] Tomai P., Lippiello A., D’Angelo P., Persson I., Martinelli A., Di Lisio V., Curini R., Fanali C., Gentili A. (2019). A low transition temperature mixture for the dispersive liquid-liquid microextraction of pesticides from surface waters. J. Chromatogr. A.

[12] Tomai P., Gentili A., Curini R., Gottardo R., Tagliaro F., Fanali S. (2021). Dispersive liquid-liquid microextraction, an effective tool for the determination of synthetic cannabinoids in oral fluid by liquid chromatography–tandem mass spectrometry. J. Pharm. Anal..

[13] Xu F., Li Q., Wei W., Liu L., Li H. (2018). Development of a liquid–liquid microextraction method based on a switchable hydrophilicity solvent for the simultaneous determination of 11 drugs in urine by GC–MS. Chromatographia.

[14] Xu F., Liu L. (2019). Simultaneous determination of free methamphetamine, pethidine, ketamine and tramadol in urine by dispersive liquid–liquid microextraction combined with GC–MS. Forensic Sci. Res..

[15] Alahyari E., Setareh M., Shekari A., Roozbehani G., Soltaninejad K. (2018). Analysis of opioids in postmortem urine samples by dispersive liquid-liquid microextraction and high performance liquid chromatography with photo diode array detection. Egypt. J. Forensic Sci..

[16] Xin L.I.U., Rao F.U., Min L.I., Li-Ping G.U.O., Li Y.A.N.G. (2013). Ionic liquid-based dispersive liquid-liquid microextraction coupled with capillary electrophoresis to determine drugs of abuse in urine. Chin. J. Anal. Chem..

[17] Mercieca G., Odoardi S., Cassar M., Rossi S.S. (2018). Rapid and simple procedure for the determination of cathinones, amphetamine-like stimulants and other new psychoactive substances in blood and urine by GC–MS. J Pharm. Biomed. Anal..

[18] Kohler I., Schappler J., Sierro T., Rudaz S. (2013). Dispersive liquid–liquid microextraction combined with capillary electrophoresis and time-of-flight mass spectrometry for urine analysis. J. Pharm. Biomed. Anal..

[19] Airado-Rodríguez D., Cruces-Blanco C., García-Campaña A.M. (2012). Dispersive liquid–liquid microextraction prior to field-amplified sample injection for the sensitive analysis of 3, 4-methylenedioxymethamphetamine, phencyclidine and lysergic acid diethylamide by capillary electrophoresis in human urine. J. Chromatogr. A.

[20] Farajzadeh M.A., Mogaddam M.R.A., Aghanassab M. (2016). Deep eutectic solvent-based dispersive liquid–liquid microextraction. Anal. Methods.

[21] Shishov A., Bulatov A., Locatelli M., Carradori S., Andruch V. (2017). Application of deep eutectic solvents in analytical chemistry. A review. Microchem. J..

[22] Gallo V., Tomai P., Gherardi M., Fanali C., De Gara L., D’Orazio G., Gentili A. (2021). Dispersive liquid-liquid microextraction using a low transition temperature mixture and liquid chromatography-mass spectrometry analysis of pesticides in urine samples. J. Chromatogr. A.

[23] Dal Bosco C., Di Lisio V., D’Angelo P., Gentili A. (2021). Hydrophobic eutectic solvent with antioxidant properties: Application for the dispersive liquid-liquid microextraction of fatsoluble micronutrients from fruit juices. ACS Sustain. Chem. Eng..

[24] Fernandez L., Silva L.P., Martins M.A.R., Ferreira O., Ortega J., Pinho S.P., Coutinho J.A.P. (2017). Indirect assessment of the fusion properties of choline chloride from solid–liquid equilibria data. Fluid Phase Equilib.

[25] Omotola E.O., Olatunji O.S. (2020). Quantification of selected pharmaceutical compounds in water using liquid chromatography-electrospray ionisation mass spectrometry (LC-ESI-MS). Heliyon.

[26] Pearson M.A., Lu C., Schmotzer B.J., Waller L.A., Riederer A.M. (2009). Evaluation of physiological measures for correcting variation in urinary output: Implications for assessing environmental chemical exposure in children. J. Expo. Sci. Environ. Epidemiol..

[27] De Araújo W.R., Salles M.O., Paixão T.R. (2012). Development of an enzymeless electroanalytical method for the indirect detection of creatinine in urine samples. Sens. Actuators B Chem..

[28] (2001). Guidance for Industry: Bioanalytical Method Validation.

