# Supplementary material for: Application of a Low Transition Temperature Mixture for the Dispersive Liquid–Liquid Microextraction of Illicit Drugs from Urine Samples
Source: Molecules. 2021 Aug 28;26(17):5222. doi: 10.3390/molecules26175222 (PMC8434511; doi:10.3390/molecules26175222)
Supplement: Supplementary file 1 [file molecules-26-05222-s001.zip › molecules-1328004-supplementary.pdf]

Application of a low transition temperature mixture for the dispersive liquid-liquid microextraction of illicit drugs from urine samples.

Valeria Gallo[1], Pierpaolo Tomai[1], Valerio Di Lisio[1], Chiara Dal Bosco[1], Paola D'Angelo[1], Chiara Fanali[2], Giovanni D'Orazio[3], Ilaria Silvestro[1], Yolanda Picó[4], Alessandra Gentili[1*]

[1] Department of Chemistry, Sapienza University of Rome, P.le Aldo Moro 5
[2] Unit of Food Science and Nutrition, Department of Science and Technology for Humans and the Environment, Università Campus Bio-Medico di Roma, via Álvaro del Portillo 21, 00128 Rome, Italy.
[3] Institute for the Biological Systems, National Research Council, Via Salaria km 29,300 00015 Monterotondo (RM)
[4] Environmental and Food Safety Research Group of the University of Valencia (SAMA-UV), Desertification Research Centre (CIDE), CSIC-GV-UV, Moncada-Naquera Road 4.5 km, 46113 Valencia, València, Spain

* Correspondence: alessandra.gentili@uniroma1.it; Tel.: +390649693230

Table S1. Structures, exact masses and main physicochemical characteristics of the selected illicit drugs

Analyte	Monoisotopic Mass	pKa	log P	Structure
BUF	204.12626	9.91	1.29	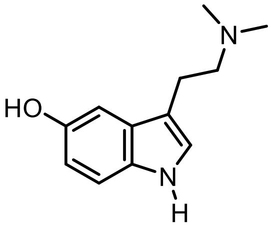
KET	237.09204	7.45	3.35	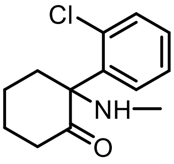
MDPPP	247.12084	6.80	2.02	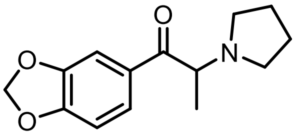
MPBP	231.16231	7.70	3.43	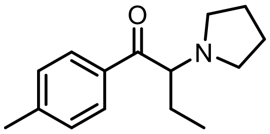
α-PVP	231.16231	7.90	3.36	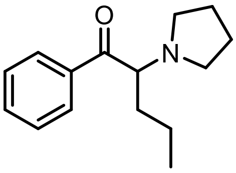
HER	369.15762	7.95	1.58	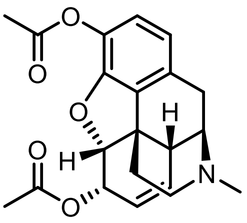

COD	299.15214	8.21	1.19	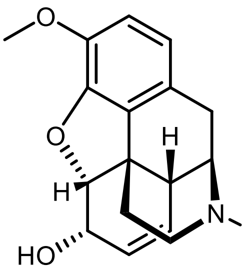
MDMA	193.11028	10.14	1.86	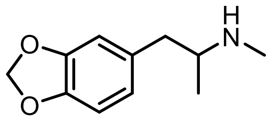
PMA	165.11536	10.00	1.65	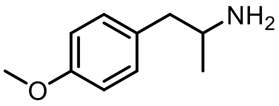
EPH	165.11536	9.52	1.13	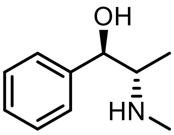
bk-MMBDB	235.12084	7.10	2.14	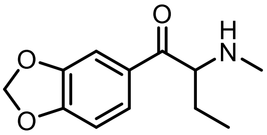
MBDB	207.12592	10.30	2.38	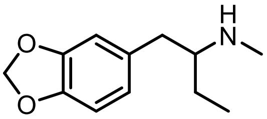
MDPV	275.15214	7.40	2.99	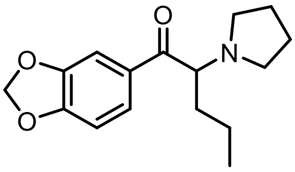
NAPH	281.17796	8.00	4.35	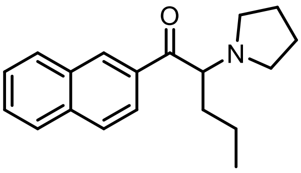

Table S2. Conditions for the preparation of some mixtures based on choline chloride and sesamol

**Components**		**Molar ratio**	**Temperature of preparation (°C)**	**Time of preparation (min)**
**Hydrogen-Bond Acceptor (HBA)[a]**	**Hydrogen-Bond Donor (HBD)[b]**			
Choline chloride	Sesamol	1:1	50	10
		1:2	50	5
		1:3	50	5
		1:4	50	10

[a] Melting point of ChCl: 302◦C. [b] Melting point of Sesamol: 62°C.
